# A Decade of Progress in Ultrasound Assessments of Subcutaneous and Total Body Fat: A Scoping Review

**DOI:** 10.3390/life15020236

**Published:** 2025-02-05

**Authors:** Monica Neagu, Adrian Neagu

**Affiliations:** 1 Department of Functional Sciences, “Victor Babeș” University of Medicine and Pharmacy of Timișoara, E. Murgu Sq, No. 2, 300041 Timisoara, Romania; neagu.monica@umft.ro; 2 Center for Modeling Biological Systems and Data Analysis, “Victor Babeș” University of Medicine and Pharmacy of Timișoara, E. Murgu Sq, No. 2, 300041 Timisoara, Romania; 3 Department of Physics and Astronomy, University of Missouri, Columbia, MO 65211, USA

**Keywords:** body composition, body fat distribution, adipose tissue, adiposity, ultrasonography, anthropometry

## Abstract

Body composition assessment by ultrasonography is a vivid research field. Ultrasound (US) can be used to quantify subcutaneous and visceral fat, to evaluate the quantity and quality of skeletal muscle, and to infer intracellular fat content. This scoping review aimed to summarize recent advancements in subcutaneous fat estimation using US and related applications. A systematic search was conducted on PubMed, MEDLINE, Scopus, Google Scholar, and Web of Science to identify original articles published in English between 1 January 2014 and 20 December 2024. A total of 1869 articles were screened based on their titles and abstracts, and 283 were retrieved for full-text evaluation. Our search and selection strategy resulted in 89 eligible documents. The literature discussed in this review suggests that US is a reliable and valid technique for measuring subcutaneous fat thickness at selected anatomic locations. Standardized measurement protocols enabled accurate subcutaneous adipose tissue (SAT) patterning in various populations (e.g., athletes, children, adults, and patients with anorexia nervosa). Further research is warranted to establish clinically relevant cutoff values. US-derived SAT thicknesses can also provide whole-body fat estimates of fat mass (FM), fat-free mass (FFM), and body fat percentage (%BF). To this end, prediction formulas were developed to ensure agreement with criterion measures given by laboratory techniques, or multicompartment models based on combinations thereof. The resulting assessments of global adiposity were reliable but inaccurate in certain populations (e.g., overweight and obese). Nevertheless, due to its high reliability, US might be used to track changes in body fat content during nutritional and/or lifestyle interventions. Future investigations will be needed to evaluate its accuracy in this respect and to improve the validity of whole-body fat estimation compared to multicompartment models.

## 1. Introduction

Recent trends in body composition research focus on phenotyping people for global and regional adipose tissue mass as well as skeletal muscle mass. To this end, a wide range of techniques have been developed and their combination provides an accurate assessment of body components at the atomic, molecular, cellular, tissue/organ, and whole body levels [1]. At the molecular level, the simplest model divides the human body in two components: body fat (primarily triglycerides) and fat-free constituents. In this two-compartment (2C) model, the task is to measure the fat mass (FM) or the fat-free mass (FFM).

Monitoring FM and FFM, and not just body mass (BM) or the body mass index (BMI), is essential for effective body weight management. Caloric restriction leads to a decrease in both FM and FFM, in variable proportions [2,3]. Furthermore, as glucagon-like peptide-1 (GLP-1) receptor agonists induce weight loss as effectively as bariatric surgery, they raise concerns about their side effects on muscle mass and function [4]. According to randomized clinical trials, GLP-1-based therapies resulted in lean mass loss ranging from 40% to 60% of the total decrease in BM. Semaglutide, for example, determined a loss of lean mass of 45.2% of the total weight loss in non-diabetic obese subjects [5]. Body composition assessment is crucial also in sports medicine. In weight-sensitive or aesthetic sports, athletes often adopt abnormal eating habits to reduce FM and maintain FFM at a level thought to optimize competitive performance [6]. Healthy eating and exercise is occasionally interrupted by extreme dieting and/or training, dehydration, and use of purging medication. These can result in eating disorders, low bone density, and less than essential body fat. While it is a mechanically inactive ballast, the adipose tissue is also an important endocrine organ [7]. The Medical Commission of the International Olympic Committee (IOC) seeks to develop guidelines to mitigate the health risks of weight management in athletes, and body composition tracking is an essential component therein [8].

Body fat content can be measured using densitometry, whereby BM and body volume (BV) are measured, and body density (D = BM/BV) is calculated. Then, body fat percentage, %BF = (FM/BM) × 100%, can be expressed in terms of body density. The underlying assumption is that, at least in certain populations (age, race, or ethnic groups), inter-individual differences in fat and fat-free body densities are negligible [9,10]. This assumption is supported by cadaver studies. In young sedentary men, for instance, Siri found that body fat had a density of 0.9 g/mL, whereas the fat-free body had a density of 1.1 g/mL; consequently, he expressed percent body fat as follows [11]: %BF = (4.95/D − 4.50) × 100%. Known as the Siri equation, this formula was validated also for non-obese adult women [12]. Fat-free body density was found to evolve during growth, mainly because of changes in hydration; therefore, in children and youth, the relationship between %BF and D depends on sex and age [12]. The observed racial differences in the composition of the fat-free body resulted in race-specific equations for %BF (see, e.g., ref. [13] and references therein).

Body volume can be determined through non-invasive methods such as hydrostatic weighing (HW) [14] and air displacement plethysmography (ADP) [15,16]. The latter is preferred due to its speed and convenience. HW has traditionally served as the gold standard for densitometry, and ADP has been calibrated to align closely with HW [16]. Densitometry is also used in conjunction with other techniques to assess various aspects of body composition. For instance, the four-compartment (4C) model utilizes ADP or HW to measure body volume, deuterium dilution or bioelectrical impedance spectroscopy (BIS) to determine the total mass of water in the body, and dual-energy x-ray absorptiometry (DXA) to assess bone mineral content [17].

The most accurate assessment of human body composition is based on laboratory techniques. They require expensive equipment, adequate space, and trained personnel [18]. These are beyond reach in large-scale studies of the nutritional status, in clinical assessments of patients with metabolic disorders, or in event-site tests of the body composition of athletes.

Hence, in practice, portable and affordable field methods are preferred. Among them, skinfold thickness measurements are the most popular [19], but bioelectrical impedance analysis (BIA) and ultrasound (US) are also increasingly used. A skinfold is formed by pinching the skin and the underlying adipose tissue between the thumb and the index finger. Then, a specifically designed mechanical caliper is used to measure the thickness of the skinfold—a double layer of skin and fat [20]. Skinfold thicknesses recorded in particular sets of anatomical locations are entered in prediction formulas to compute body density and %BF. A variety of such formulas have been proposed and validated against HW [19]. In BIA, an alternating current of low (imperceptible) intensity is passed through the subject’s body and electrical quantities are measured, such as Ohmic resistance, capacitive reactance, and impedance, which characterize the extent to which the body opposes the passage of the electric current. Then, population-specific formulas relate electrical quantities to body composition parameters [21]. US has been employed to measure subcutaneous fat thickness in humans since the 1960s [22,23]. Early attempts at using US to evaluate nutritional status were hampered by high equipment costs and produced mixed results [24,25]. Nevertheless, the emergence of low-cost instruments and dedicated software opened up new avenues in the study of body composition by US [26].

This paper is a scoping review of recent advancements in US-based evaluation of human body fat content. Although early milestones are mentioned occasionally for the sake of clarity, this article deals with developments reported since 2014 because previous work is covered by an inspirational review paper [26]. Furthermore, the scope of this paper is limited to the characterization of global adiposity and subcutaneous fat patterning. It does not discuss methods devised to evaluate visceral fat, hepatic steatosis, and intramuscular fat—the latter were addressed by excellent reviews [27,28,29]. The uses of US in the evaluation of muscle mass and quality by dietitians was presented in a recent narrative review [30]. Also, a novel methodology emerged under the name of Nutritional Ultrasound^®^, which proposes a standardized quantification of the thigh muscles and abdominal adipose tissue depots [31]. Amplitude-mode US measurements of the subcutaneous fat thickness and subsequent evaluation of full-body adiposity have been addressed by a recent systematic review [32], whereas brightness-mode US studies of the subcutaneous adipose tissue thickness were summarized in another systematic review [33]. Their format, however, asked for a well-defined, narrow research question and strict quality assessment of the included original works. Therefore, we chose to write a scoping review to map the field of US assessment of subcutaneous and whole-body fat in adults and to identify research gaps [34,35].

The remainder of this article is organized as follows: first, we present basic concepts of ultrasonography; then, we describe the search methodology; next, we summarize the eligible papers; and finally, we discuss the perspectives of the field.

## 2. Basics

Humans can hear sound within the range of 20 Hz to 20 kHz; ultrasounds are not audible because their frequencies exceed 20 kHz. Medical US imaging is performed at MHz frequencies as a result of a trade-off between two opposite requirements regarding resolution and range of view—the higher the frequency, the higher the image resolution ([36], Ch. 3) but lower the penetration depth [37].

Ultrasound transducers incorporate piezoelectric ceramics, which generate US waves when subjected to oscillating electric signals—a phenomenon known as the inverse piezoelectric effect. For imaging, a short US wave (pulse) is delivered into the body. It travels at a speed c (the speed of sound, which depends on tissue type, pressure, and temperature [38], Ch. 5) until it encounters a surface of separation between two media that differ in their acoustic impedance, Z=ρc, where ρ denotes density. If the interface is perpendicular to the wave’s direction of propagation, part of the pulse returns along the same direction. The intensity of the reflected wave (energy transported through the unit area during one second) is a fraction of the intensity of the incoming wave, given by ${\left(Z_{2}-Z_{1}\right)}^{2}/{\left(Z_{2}+Z_{1}\right)}^{2}$, where $Z_{1}$ and $Z_{2}$ are the acoustic impedances of the two media ([36], Ch. 2). To conduct a US scan, a small amount of gel is applied to the transducer to facilitate the passage of US waves into the body. (Due to its extremely small acoustic impedance, even a thin layer of air would cause strong reflections.)

As the US pulse encounters tissue interfaces (e.g., fat–muscle, muscle–bone), parts of it are reflected back as echoes, with the transducer serving both to emit the US pulse and capture the returning echoes. These cause vibrations in the transducer, which are converted into electric signals through the piezoelectric effect and processed by the instrument. From the time, Δt, passed between launching the pulse and receiving the echo, one calculates the distance, d, from the transducer to the tissue interface, d=cΔt/2. To compute the depth from which an echo originates, US scanners conventionally use an estimated average of the speed of sound in biological soft tissues, *c* = 1540 m/s ([36], Ch. 2).

The resolution of US imaging is of the order of one wavelength, λ=c/f, where f stands for the frequency ([36], Ch. 3). US scanners work at frequencies that cover one order of magnitude, from 2 to 25 MHz, corresponding to resolutions ranging from 0.8 mm to 0.06 mm, respectively.

In an amplitude (A)-mode US, the intensities of echoes are plotted versus the depths from which they originate. In a brightness (B)-mode US, a transducer comprising an array of piezoelectric elements records intensities and depths to create an image in which pixel values represent intensity: the more intense an echo, the lighter the corresponding pixel. Hence, in a B-mode US image, echogenic interfaces appear as bright stripes.

To test individual anatomic sites, like those used in subcutaneous fat thickness measurements by an A-mode US, the transducer is slid back and forth about ± 5 mm from the target site to ensure a spatial averaging of the US signal. Throughout the entire measurement, the operator should ensure that the transducer is perpendicular to the skin and does not deform the underlying tissues. The operation of a particular A-mode US scanner specifically designed for body composition studies is presented in detail in [26].

For a B-mode US scan, a thick (3–5 mm) layer of gel is placed between the transducer and the skin. The transducer is held in contact with the gel, but away from the skin; thereby, no inward force is exerted on the body. Remarkably, after a brief instruction, operators with no previous experience in ultrasonography were capable of recording US images for a reliable determination of the subcutaneous adipose tissue (SAT) thickness [39].

Since B-mode scanners are usually operated at high frequencies, of the order of 10 MHz, their axial resolution is of about 0.1 mm. Thus, the gel layer can be distinguished as a dark band on the top of the US image, and the skin can be observed along with fibrous structures (fascia) embedded in subcutaneous tissues. Nevertheless, interpreting the scan can be challenging and subjective. Different tissue interfaces, like skin–fat, fat–muscle, and muscle–bone, appear as continuous bands of bright pixels. However, fascia can also appear as light streaks, potentially leading to misinterpretation. It takes a skilled observer to spot tissue interfaces (e.g., the fat–muscle interface) and measure the thickness of the tissue layer of interest. To expedite image analysis, a dedicated software has been developed for a semi-automatic measurement of the SAT thickness [39,40,41].

## 3. Methods

We conducted this scoping review according to the guidelines published in the Preferred Reporting Items for Systematic Reviews and Meta-Analyses (PRISMA) 2020 statement [42]. More precisely, we followed the reporting guidance given in the PRISMA extension for scoping reviews and the PRISMA-ScR-Fillable-Checklist [43].

### 3.1. Eligibility Criteria

Studies included in this review needed to meet all of the following inclusion criteria: original articles published between 1 January 2014 and 20 December 2024, written in English, reported measurements of the SAT thickness in human subjects using A-mode or B-mode US, and sought to characterize nutritional status or estimate the amount of whole-body fat.

Papers were not considered if they met any one of the following exclusion criteria: reported visceral fat assessments, focused on muscle thickness and/or quality, evaluated intracellular fat content, or reported the results of in vitro or animal studies.

### 3.2. Information Sources and Search Strategy

We conducted a systematic search on PubMed, MEDLINE, Scopus, Google Scholar, and Web of Science to identify potentially relevant articles. A title and abstract search was performed for all the databases except for Google Scholar, which was restricted for title search only to keep the number of hits manageable. The search methodology was devised iteratively by each researcher and refined through discussions between November 2023 and March 2024. The final search terms are presented in the Supplementary Materials, Table S1. The search was repeated monthly up until 20 December 2024.

### 3.3. Document Selection and Data Collection

We exported the search results into Excel (Microsoft Corporation, Redmond, WA, USA) and EndNote X7.0.1 (Clarivate Analytics, Philadelphia, PA, USA). Duplicates of documents with a digital object identifier (DOI) were removed using Excel, whereas in the absence of DOI, duplicates were identified and removed manually.

**Figure 1:**
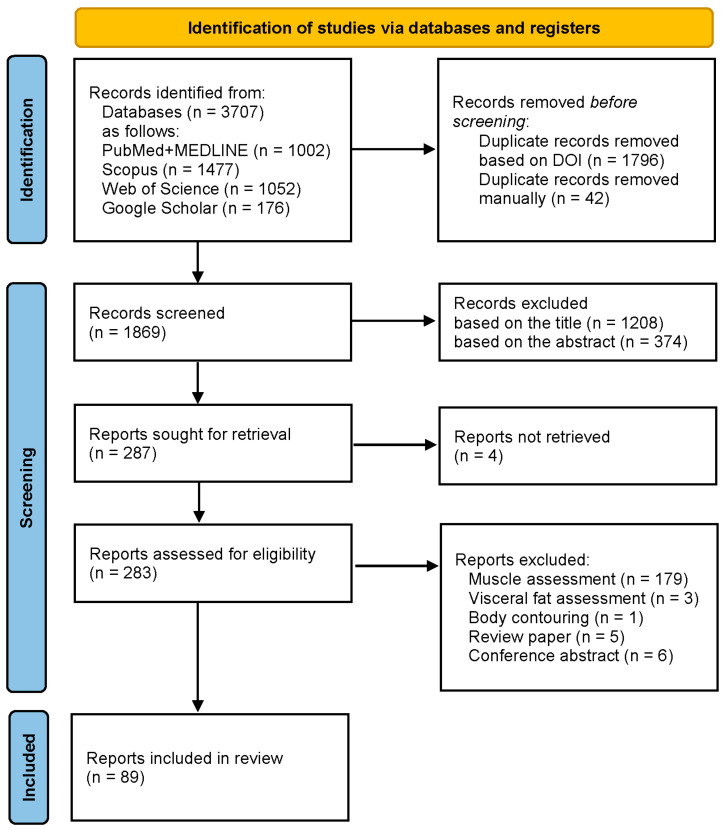
The PRISMA 2020 flow diagram of document search and selection.

The PRISMA 2020 flow diagram of document handling is shown in Figure 1 [44].

Each record was screened by two reviewers independently and the lists of excluded records were discussed before a final decision was made. Retrieved reports were handled in a similar manner: the eligibility of each full text was evaluated by the reviewers on their own and discussed in periodic meetings. We also scrutinized the bibliography of each included article to look for relevant works but did not find further eligible reports. No automation tools were used in the screening process.

We first agreed on the data collection methodology. Then, we extracted the relevant data independently, and saved them in Excel spreadsheets. The findings were compared in meetings to ensure the accuracy and completeness of the collected data.

Regarding the validity of US for SAT thickness measurements and total body fat assessments, we sought to extract the following statistical measures of accuracy: the standard error of estimate (SEE), the total error (TE), the constant error (CE) defined as the mean difference between the compared methods (US minus the criterion measure), and the limits of agreement (LoA)—the latter were identified from Bland–Altman analyses [45]. Concerning reliability, we looked for the standard error of measurement (SEM), minimal detectable change (MDC), and intraclass correlation coefficient (ICC) [46].

## 4. Results

The literature included in this scoping review provided an overview of the field. Before diving into details, we briefly describe the main approaches to the evaluation of subcutaneous and total fat content of the human body.

One option is to measure the SAT thickness at well-defined sites and compute the sum of the measured thicknesses. Another option is to use regression equations to predict body density or %BF based on certain SAT thicknesses. The latter responds to the common desire of clinicians and coaches to evaluate global adiposity in terms of total FM or %BF, but also raises concerns that the prediction equations add an extra layer of complexity and potential sources of error [19]. Just as in anthropometry, a variety of prediction formulas were established for diverse populations against different criterion methods. Therefore, certain investigators argued against converting SAT thicknesses into %BF [19], while others sought to identify the most accurate way to compute %BF in specific populations based on US-derived data [47,48,49,50,51].

**Figure 2:**
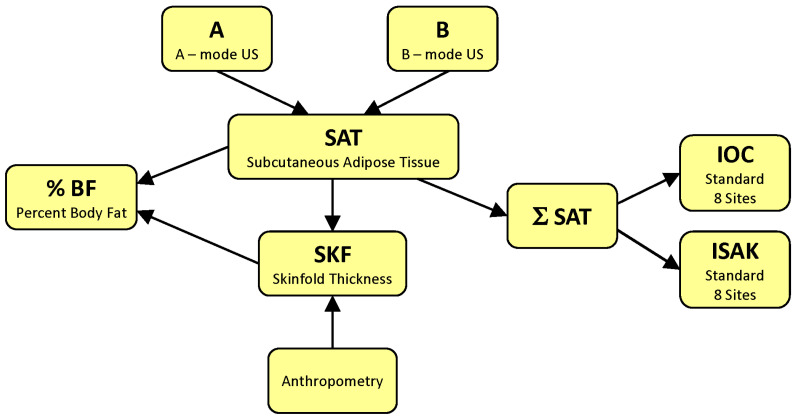
Block diagram of directions of research in US-based evaluation of body fat content. Abbreviations: IOC—International Olympic Committee; ISAK—International Society for the Advancement of Kinanthropometry.

Figure 2 represents a block diagram of major strategies aimed at quantifying body fat by ultrasonography.

The most common applications of US for the analysis of human body fatness seek to determine %BF. To this end, one strategy is to measure SAT thickness via an A-mode US at specific sites and use prediction formulas to compute %BF (i.e., to follow the A→SAT→%BF pathway on the flow diagram from Figure 2) [26]. Another is to acquire B-mode US images and measure the SAT thickness using electronic calipers [52] or an image processing and segmentation software [39], and then compute %BF using equations obtained via multiple regression analysis of SAT thicknesses compared to a criterion method (B→SAT→%BF) [52]. Alternatively, prediction formulas for %BF can be derived from anthropometric equations provided that an accurate relationship has been established between SKF and SAT (B→SAT→SKF→%BF) [53].

Other strategies consist in quantifying body fat content by sums of SAT thicknesses. The selection of relevant sites may rely on previous experience with anthropometry. In this case, one can characterize a person’s body fat content by computing the sum of SATs measured, for instance, at standard ISAK sites [54] (B→SAT→∑SAT→ISAK or A→SAT→∑SAT→ISAK) [19,55,56]. Alternatively, one can take US scans at the sites identified by the IOC Working Group on Body Composition, Health and Performance [41], which allow for accurate and reliable measurements of the SAT thickness (e.g., B→SAT→∑SAT→IOC) [41,57,58] or A→SAT→∑SAT→IOC [59]).

### 4.1. Ultrasound Measures of the Subcutaneous Fat Layer Thickness

US-based measurements of SAT thickness have a long and sinuous history [26]. They have gathered momentum recently due to the emergence of highly reliable US scanning methods. For example, Toomey et al. [60] published a seminal study aimed at evaluating the dependence of the measured SAT thickness on the force exerted by the transducer on the body, and the orientation of the transducer with respect to the underlying muscles—transverse or longitudinal. A set of seven ISAK sites were marked using a surgical pen and a wound closure strip was placed over the site to make sure that the US image represents the tissues located right beneath the mark. SAT thickness was measured, via electronic calipers, as the distance between the inferior border of the dermis and the upper border of the fat–muscle interface. The compression force was varied from 0.5 N (the minimum needed for image formation) to about 10 N (referred to as maximum force, beyond which no further deformation was observed). The measured SAT thickness decreased non-linearly with the applied force by at most 36% at the triceps site, 37% at the abdominal site, and 25% at the front thigh site. These results imply that the operator should apply the least possible force to prevent tissue compression. In the B-mode US, the necessary force can be tuned on-the-fly by inspecting the on-screen image. Unlike the applied force, transducer orientation induced low variability regardless of site [60].

**Figure 3:**
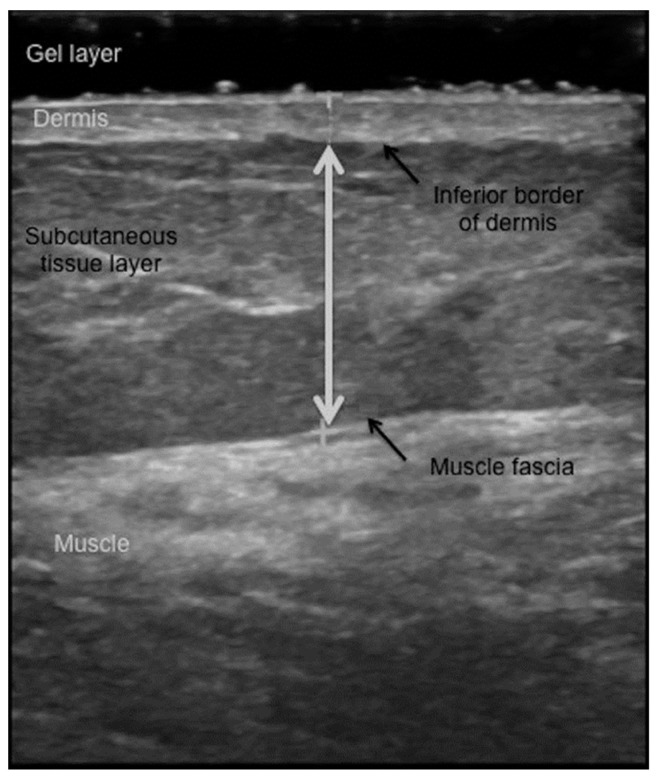
Measurement of the SAT layer thickness on the high-resolution US image of the front thigh (reprinted from [63] with permission from Georg Thieme Verlag KG).

This procedure was adopted by most subsequent studies of subcutaneous adiposity via the B-mode US. Figure 3 shows a sonogram of soft tissues beneath the skin and illustrates the measurement of the thickness of the SAT (also known as subcutis). A thick layer of hydrogel is placed over the target site to minimize the inward force; it appears as a dark band on the top of the sonogram. The SAT thickness is measured, using electronic calipers, as the distance between the dermo-hypodermal junction and the most superficial layer of the deep fascia. The dermo-hypodermal junction (inferior border of the dermis) can be distinguished as a single hyperechoic line (Figure 3, upper black arrow). The deep fascia, which covers the muscle, is visible as a multilayered echogenic band; the most superficial one corresponds to the bottom border of the SAT (Figure 3, lower black arrow). The histological and sonographic features of subcutaneous soft tissues are thoroughly described by Ricci et al. [61]. The bright lines visible on the US image of the subcutis originate from its fibrous scaffold. The major one stems from the superficial fascia, which separates the superficial subcutis (a honeycomb-like arrangement of large fat lobules separated from each other by fibrous connective tissue) from the deep subcutis (smaller fat lobules hosted by a less elastic scaffold). These structural elements suffer changes in certain pathological conditions such as lipedema or lymphedema, and US reveals them [62].

Over the past decade, we have witnessed a revival of the B-mode US applied for SAT measurements due to the works of Müller et al. [39,40]. They used B-mode US scanners, working at 12 MHz and 11.2 MHz, to visualize subcutaneous tissues of athletes at eight standard anthropometric sites recommended by the ISAK protocol: biceps, triceps, subscapular, iliac crest, supraspinale, abdominal, front thigh, and medial calf [54]. Later on, they proposed another set of sites to boost the precision and accuracy of SAT measurements; these will be discussed on detail in Section 4.4.

Another option for SAT thickness measurements is the A-mode US. It elicited much interest recently [32], as user-friendly instruments became available at moderate prices [26].

**Figure 4:**
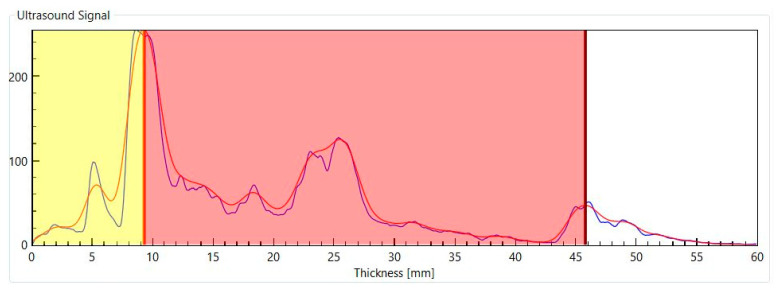
Measurement of the SAT layer thickness at the front thigh using an A-mode US. The yellow portion of the plot corresponds to the subcutaneous fat (skin included), whereas pink represents the anterior part of the quadriceps muscle at mid-thigh (the peak within the pink region stems from the fascia that separates rectus femoris from vastus intermedius). This image is a screen capture of the BodyView ProFit software (version 2.1.0.9045) taken in our laboratory.

An A-mode US scan is a plot of the intensity of the reflected US waves versus the depth from which they originate (Figure 4).

The A-mode scan shown in Figure 4 has been generated using the BodyMetrix system (IntelaMetrix, Livermore, CA, USA), which works at a frequency of 2.5 MHz [64]. Its axial resolution is insufficient to resolve the skin borders, and therefore, the SAT thickness is defined as the distance between the transducer head and the fat–muscle interface; the latter appears as the first major peak in the scan. The subcutaneous fat layer corresponds to the yellow shaded part of the plot [65].

The accuracy of A-mode US measurements of the SAT thickness was investigated in a recent study by Wagner et al. [66]. A-mode and B-mode US measures of the SAT thickness were acquired on six cadavers at the abdomen, thigh, triceps, calf, suprailiac (only in women), and chest (only in men). Then, the cadavers were dissected and the SAT thickness was measured at each site using the ruler of a digital caliper. At most sites, the three methods provided mean values within 1 mm. A notable exception was the suprailiac site, at which the technician was unable to interpret the B-mode scan. Two-way repeated measures ANOVA revealed no significant effect for method, site, or their interaction [66].

When A-mode and B-mode US were compared by measuring SAT thickness at seven sites [67], equivalence testing with an interval of ±1 mm indicated no significant mean difference between the two techniques for all but the abdominal site. Greater variability was observed at the trunk compared to the limbs. Based on these results, and their previous cadaver validation study [66], the authors concluded that A-mode and B-mode US provide similar measures of the SAT thickness. In a recent study conducted with a different A-mode US device, Lee et al. reached the same conclusion: the mean difference between the SAT and muscle thicknesses determined by the low-resolution A-mode US and the high-resolution B-mode US was less than 0.14 mm, and the ICCs exceeded 0.95 [68].

### 4.2. A-Mode Ultrasound Assessments of the Total Body Fat Content

The BodyMetrix instrument comes with a dedicated software, called BodyView, which provides data analysis and management tools. The manufacturer has released several flavors of BodyView (e.g., Professional, ProFit, Personal), which differ in design and capabilities, but all of them offer multiple options to compute %BF based on SAT thicknesses measured at certain sites. The underlying proprietary formulas have been inspired by anthropometric equations that express body density in terms of SKF thicknesses measured at the same sites. In anthropometry, the most popular ones are the 3-site and 7-site Jackson and Pollock equation devised for men [69], and the Jackson, Pollock, and Ward equation developed for women [70]; regardless of sex, they are commonly abbreviated as JP3 and JP7, respectively. Once body density is obtained, %BF can be computed using the Siri equation [11] or its alternatives developed for specific populations.

Establishing the validity (accuracy) and reliability (precision) of %BF assessments by the BodyMetrix and its associated software have attracted much interest over the past 10 years. In the remainder of this section, we discuss representative studies of the accuracy and reliability of an A-mode US. Further details on this topic are given in a thorough narrative review [71] and a recent systematic review and meta-analysis [32].

#### 4.2.1. Validity of Body Fat Content Estimates via A-Mode Ultrasound

The term validity, or accuracy, refers to the ability of a technique to provide the true value of the measured quantity. In the most common case when the true value is unknown, testing the accuracy amounts to compare the obtained results with those given by a trusted reference technique, which provides a criterion measure.

The BodyMetrix was found accurate compared to HW in a study conducted on male high school wrestlers [72]. FFM estimates did not differ significantly between the two techniques, and the standard error of estimate (SEE) was 2.4 kg. In the Bland–Altman (BA) analysis [45,73], the mean difference, also known as constant error (CE), was 0.2 kg, and the limits of agreement (LoA) were approximately −4.5 kg and 4.9 kg [72]. This early success motivated further studies, turning the BodyMetrix into the most investigated A-mode US instrument developed so far for body composition analysis.

Table 1 presents the most important findings of validation studies published between 2014 and 2024; listed are sample characteristics (mean ± standard deviation) and statistical measures of accuracy.

In their investigation conducted on female collegiate gymnasts [74], Loenneke et al. found that, compared to DXA, on average, the BodyMetrix overpredicted %BF by 3.4% BF via the JP3 formula and by 5.7% BF via the one-point biceps (Bic1) formula.

The validation study of Smith-Ryan et al. focused on overweight and obese subjects evaluated by the BodyMetrix instrument in conjunction with the JP7 formula [75]. As a reference method, they used Siri’s 3C model [11], which relies on measurements of BV and total body water—TBW (kg); BV was determined by ADP and TBW was inferred from BIS. Despite its simplicity, Siri’s 3C model is highly appreciated in the literature because it is insensitive to variations in fat-free mass hydration and demonstrates excellent agreement with 4C, 5C [90], and 6C [91] models. In comparison with the 3C model, US underestimated the total amount of body fat: the CE was −4.7% BF for the entire sample, −4.2% BF for the overweight participants (*n* = 27), and −5.2% BF for the obese subjects (*n* = 20).

The progressive underestimation of %BF by the JP7 formula in people with high body fat content was observed also in [49], which evaluated the BodyMetrix against ADP. In a heterogeneous sample from the general population, this tendency was apparent in both women and men. In the BA analysis, proportional bias was present regardless of sex. The least squares regression line of differences vs. means intersected the line of identity at about 18% BF for women and 12% BF for men, suggesting that the JP7 formula from BodyView has been optimized to give accurate assessments in lean individuals.

Hendrickson et al. [76] reported that, in a group of normal-weight adults, the A-mode US with the JP3 protocol underpredicted the amount of body fat by about 1% BF with respect to ADP. Totosy de Zepetnek et al. found that the mean difference between the JP7 protocol and ADP was −0.3% BF in a mainly normal-weight, racially heterogeneous sample of the general population [77]. Along the same lines, Johnson et al. reported very good agreement between JP7 and ADP in a group of college-aged adults [79] (Table 1).

Wagner et al. tested the validity of the BodyMetrix in collegiate athletes [51]. They estimated %BF using the JP3 formula and found mean differences of about 1.5% BF in men and 4.7% BF in women, concluding that the JP3 formula was in good agreement with ADP for males, but not for females. In elite male rowers, Kendall et al. observed a significant overestimation of the FFM compared to the 4C model. Nevertheless, on average, the BodyMetrix-derived FFM was within 0.9 kg from ADP [92].

The comparative evaluation of the JP3, JP7, Bic1, and 9-site Parrillo (P9) protocols for the body composition assessment of male soccer players [93] demonstrated that JP3 and JP7 were equally valid, whereas the other two protocols overestimated %BF in male athletes: compared to JP7, the mean difference was 3.1% BF for Bic1 and 4.7% BF for P9.

Johnson et al. explored the accuracy of the BodyMetrix using the JP7 formula in normal-weight young adults [78]. Paired sample *t*-tests showed significant differences between %BF measured by US and DXA, regardless of sex. On average, JP7 underestimated the global adiposity by 3.7% BF in women and 4.4% BF in men. Therefore, the authors concluded that the BodyMetrix using the JP7 formula might not be an accurate method for assessing %BF in normal-weight people aged between 18 and 35 years [78].

In a convenience sample of 76 college-aged participants (43F, 33M), Baranauskas et al. performed a validation study of the BodyMetrix using the JP7, JP3, and the 3-site Pollock (P3) equation with respect to DXA. All three US estimates remained below the reference, with CEs ranging from −3.6% to −5.2% BF [80]. In middle-aged subjects with higher body fat content, JP3 was even less accurate: the sample mean of %BF was 19.4% for the BodyMetrix and 29% for DXA [81].

DXA was chosen as a reference technique also by Kang et al. [82] to test the accuracy of all the options offered by the BodyView software for male college students. In their study, the BodyMetrix was used with nine prediction formulas and the agreement with DXA was evaluated using mean absolute percent errors, repeated measures analysis of variance (ANOVA), BA analysis, and equivalence testing. In agreement with previous investigations [78,80], JP3 and JP7 were found to underestimate %BF (albeit to a larger extent, with CEs of 7% and 6.4% BF, respectively). Furthermore, proportional bias was observed, just as in [49], so the disagreement between the US and reference technique was larger in the case of subjects with higher body fat content. The tendency of progressive underprediction of %BF was a common feature of all the options except for Bic1 and 4-site Forsyth–Sinning (FS4) [82]. The 4-site Durnin and Womersley (DW4) and P9 protocols (CE of −1.3% and −0.4% BF, respectively) had the 90% confidence intervals of their means within the equivalence zone (mean ± 10% of the mean given by DXA); thus, they were deemed equivalent to DXA in the case of normal-weight male college students.

Lowry et al. evaluated the accuracy against ADP of all nine formulas provided for adult men in the BodyMetrix system [48]. Their results suggest that the JP3, JP7, and NHCA 4-site formulas provide “fairly good” %BF estimates: JP3 had the lowest CE, but higher SEE than the other two competitors; JP7 had the lowest SEE and TE, but a slight systematic bias; the NHCA 4-site formula gave the highest CE of these top-three equations, but no systematic bias, and similar SEE and TE.

Regarding the discrepancy between [48,82], Lowry et al. suggested that differences between study groups (age, ethnicity, nutritional status) and reference techniques might be responsible for the disagreement [48]. Indeed, when DXA and ADP were compared to a criterion 5C model [90], DXA overestimated %BF by 1.9% or 2.9% BF, depending on whether the estimate was based on all pixels or just on soft tissue pixels, respectively (a detail not reported by Kang et al. [82]). In contrast, ADP, with the Siri equation, provided a milder overestimation of 0.9% BF. Thus, a discrepancy of about 1–2% BF could be ascribed to the different reference techniques. The observed differences, however, are larger; e.g., the P9 equation provided a mean overestimation by 4.7% with respect to ADP and an underestimation by 0.4% BF with respect to DXA. Such conflicting results underline the importance of validation studies focused on specific populations and similar criterion measures.

Ripka et al. tested the BodyMetrix with JP7 versus DXA in a sample of male adolescents and obtained significantly lower body fat estimates (Table 1) [83]. To improve the accuracy of the US, they derived a prediction equation using multivariable regression [94]: %BF = 13.281 + 0.869 × (triceps SAT + thigh SAT) − 0.252 × (age); if not specified otherwise, in this review SAT thicknesses are expressed in mm. This equation is of particular interest because limb sites allow for reliable and accurate measurements of the SAT thickness—see the next subsection. In another study [84], they investigated both sexes. As potential independent variables, they considered the SAT thicknesses involved in JP7, BM, age, and sex. These were added to the model, one term at a time, in the descending order of their correlation with %BF, and model improvement was evaluated after each step via ANOVA. This forward selection procedure came to an end when no improvement was observed. The resulting model involved only four SATs: %BF = 13.658 + 0.788 × (triceps SAT) + 0.829 × (subscapular SAT) + 0.220 × (chest SAT) + 0.479 × (thigh SAT) − 0.666 × (age) + 4.044 × (sex), where sex is a binary variable, equal to 1 for women and 0 for men [84]. Pineau and collaborators pursued a similar strategy to achieve accurate estimates of body fat content by an A-mode US in male athletes [85] as well as in a heterogeneous sample of men from the general population, aged 18–60 y [86]. Moreover, they tested their novel formulas on independent samples drawn from the same populations.

Recognizing the methodological differences between anthropometric measurements taken with skinfold calipers and SAT thickness measurements via an A-mode US, Bielemann et al. set out to improve the accuracy of %BF estimation using the BodyMetrix [87]. In particular, they took advantage of the ability of US to penetrate beyond the SAT layer and enable muscle thickness measurements. In their model, besides SAT thicknesses (mm), they also considered including muscle thicknesses (mm), limb girths (cm), height (m), BM (kg), and age (y). Stepwise linear regression analysis resulted in novel prediction equations: for women, %BF = 0.12 × (age) − 0.76 × (calf circumference) + 0.24 × (abdominal SAT) + 1.10 × (calf SAT) − 27.33 × (height) + 0.30 × BM + 67.63; for men, %BF = −0.71 × (thigh circumference) + 0.40 × (triceps SAT) + 1.01 × (thigh SAT) − 0.16 × (biceps muscle thickness) − 37.23 × (height) + 0.61 × BM + 73.23. On average, these equations overestimated body fat in comparison to ADP by merely 0.1% BF for women and 0.5% BF for men.

Bondareva and coworkers explored the relationship between %BF estimates given by the BodyMetrix via JP7 and the BIA instrument ABC-02 Medas (Medas Ltd., Moscow, Russia) [95,96]. On average, the two instruments gave similar results, but a significant proportional bias caused large differences in the case of subjects with relatively high %BF. A linear correction of BIA data eliminated the proportional bias and improved the agreement between the two methods, leading to a narrower interval of agreement. Also, Jones et al. compared the BodyMetrix, BIA, and ADP as tools for monitoring patients with short bowel syndrome. On average, US provided lower fat mass estimates than ADP [97].

Schoenfeld et al. tested the BodyMetrix using JP4 versus ADP in a group of 19 young women before and after a 4-week weight-loss intervention. The sample mean given by US was within 1% BF from ADP, and, what is most important, changes in body composition were detected with even better accuracy: while ADP recorded a mean drop of 1% BF, the change determined by US was 1.06% BF [88]. Less accurate tracking of body fat was reported by Bridge et al. in men subjected to 12 weeks of resistance training [89]. Although A-mode US (JP3) and ADP were in excellent agreement before the intervention, there was a CE of −1% BF post-intervention: the drop in %BF was 0.4% according to ADP and 1.4% according to US. The discrepancy between these works could stem from the sample characteristics, but also from one limitation admitted by Bridge et al., that subjects were allowed to drink water ad libitum before ADP testing [89].

Krkeljas et al. evaluated the BodyMetrix as a tool for estimating the resting metabolic rate [98]. They used the JP7 protocol to infer %BF and computed the resting metabolic rate via the equation implemented in the BodyView software. The Bland–Altman analysis indicated that the BodyMetrix underestimates the resting metabolic rate compared to indirect calorimetry (CE = −323 kcal/day, LoA = [−906, 260] kcal/day). When the results were split by sex, the mean underestimation was larger in women than in men (370 kcal/day and 291 kcal/day, respectively) [98].

#### 4.2.2. Reliability of %BF Estimation by A-Mode US

Reliability, or precision, refers to how close the obtained results are to each other; that is, it refers to the consistency of a measure. Ideally, a measurement technique should be both accurate and precise.

The reliability of a measurement method can be characterized by a variety of statistical quantities. These include the standard error of measurement (SEM) and minimal detectable change (MDC) [46]. The latter is of special interest because it is an estimate of the smallest difference between two scores needed to be fairly sure that they reflect a true change in the measured quantity rather than random error; MDC is also known as the minimal difference needed to be considered real [99,100]. These are known as absolute measures of reliability because they are expressed in the same units as the measured variable (e.g., % for body fat percentage and kg for FFM). The smaller they are, the higher is the reliability of the measurement. Another option is to quantify reliability in terms of intraclass correlation coefficients (ICCs), relative measures of reliability [101,102,103]. These are dimensionless quantities and the closer they are to 1 the better.

Table 2 presents essential findings reported in the literature regarding the reliability of total body fat assessments using the A-mode ultrasound.

The work of Loenneke et al. [104] reported that the Bic1 protocol was more reliable than JP3, and not just marginally, but with a twice smaller MDC (Table 2). This result is appealing from the practical point of view and reasonable, considering that limb sites allow for facile US imaging [39] (see also Section 4.4). Unfortunately, it was disproved by a more recent study conducted on a larger and more heterogeneous sample [107]. Further research will be needed to elucidate whether it holds in certain populations.

Smith-Ryan et al. [75] conducted duplicate BodyMetrix trials on overweight and obese individuals, 24 to 74 h apart. In each trial, %BF was computed according to the JP7 formula from BodyView, then FM was calculated from BM and %BF, and FFM was obtained as BM-FM. The reproducibility of US assessments of %BF and FFM was deemed acceptable, with SEMs of 2.2% BF and 1.9 kg, respectively, suggesting that US may be useful for monitoring body composition changes in overweight and obese people [75].

Hendrickson et al. [76] evaluated the reliability of the BodyMetrix with the JP3 protocol in a group of mainly normal-weight adults (BMI 23.9 ± 3 kg/m^2^, %BF 17.6 ± 6.9%). Two raters performed pairs of consecutive trials on each subject, right after one another, while being blinded to the other’s results. For the intratester reliability of %BF assessments the ICC was 0.87 for tester 1 and 0.80 for tester 2, whereas for the intertester reliability ICC was 0.87. For FFM measures, the ICCs ranged from 0.98 to 0.99.

The intra- and intertester reliability of the BodyMetrix for SAT thickness measurements and %BF assessments via the JP3 formula was investigated by Chirita-Emandi et al. on two volunteers [105]. At each anatomic site, independent measurements were taken by seven expert and eight novice raters. Remarkably, the ICCs for %BF measures were high regardless of previous experience with the BodyMetrix (0.982 for novices and 0.989 for experts), suggesting that precise results can be obtained upon minimal operator training. The intertester reliability of %BF estimates was excellent also in a group of children and adolescents between 5.3 and 18.2 years of age (Table 2) [106].

Totosy de Zepetnek et al. [77] observed excellent reliability in %BF measurements using the JP7 protocol in a group of mainly normal-weight adults (33F, 16M). Miclos-Balica et al. [107] evaluated the reliability of %BF assessment in a heterogeneous sample of the general population using JP7, JP3, P3, and Bic1. The BodyMetrix was used in automatic mode (i.e., the technicians did not overrule the SAT thickness values determined by BodyView). The measurement precision was highest for JP7 and lowest for Bic1. Remarkably, the intertester reliability of JP7 was comparable to that of ADP [108]. The excellent reliability of %BF estimates using the BodyMetrix with JP7 was confirmed by Bondareva et al. [109] in a group of 45 adults (24F, 21M) between 18 and 35 years of age: in their study, Lin’s concordance correlation coefficient, CCC, between repeated measurements was 0.99.

The highest precision recorded so far with the BodyMetrix was reported by Wagner et al. [51] (Table 2). These authors compared anthropometry, an A-mode US, and ADP in a study performed on elite collegiate athletes. SKF and US readings at the JP3 sites were taken in duplicate by expert technicians blinded to each other’s results. For consistency, the sites were marked by the first technician using a surgical marker and used thereafter. The MDC was 1.3% BF for the first technician (with 20 y of experience in anthropometry) and 1.8% BF for the second technician (6 y of experience). The ICC was 0.999 for the first technician and 0.993 for the second technician. The main factor behind the spectacular precision attained in [51] is unclear. Technician training most likely played a role. The accurate marking of the measurement sites is vital in a SKF-based assessment of body composition; a deviation of merely 1 cm from the standard sites can lead to significant errors [19]. Additionally, the physical characteristics of the study group could have influenced the reliability measures, too.

When novice examiners performed both SKF and A-mode US measurements, the intertester ICC of %BF estimations of males was significantly higher for US (0.990) than for SKF (0.862), suggesting that A-mode US requires less training than anthropometry. It is important to note, however, that a major source of error, the variability of site location, has been removed in this study (the sites marked by novice examiners were checked by experienced technicians before taking the measurements) [65].

An important question is whether US results are consistent between different testing facilities. To provide an answer, Bigler et al. evaluated the interdevice reliability of the BodyMetrix [110]. In a group of 42 men (age 28.6 ± 11.9 y) of diverse physiques (BMI 25.4 ± 4.6 kg/m^2^), the same technician measured SAT thicknesses with two different BodyMetrix instruments, at 10 sites—biceps, calf, lower back, and those involved in JP7. For SAT thicknesses, the ICC ranged between 0.939 (biceps) and 0.998 (lower back), whereas MDC was between 0.86 mm (lower back) and 1.61 mm (axilla). The mean difference between %BF values given by JP7 was 0.34 ± 1.24%, of no statistical significance (*p* = 0.09). These results demonstrate a high interdevice reliability, and thus, body fat assessments from different testing sites can be compared [110].

### 4.3. B-Mode Ultrasound Evaluations of Body Fat Content

To our knowledge, Sloan was the first to derive an equation for assessing the total amount of body fat from B-mode US images of the SAT layer [111]. In his sample of 50 young men, the thigh SAT had the highest correlation with the body density measured using HW. When two sites were considered in the multiple regression analysis, thigh and iliac crest gave the best correlation.

Abe et al. [112] developed a US-based equation for body fat estimation in Japanese adults in accord with HW. They proposed nine sites to sample the anterior and posterior parts of the trunk and limbs. Along the same lines, but relative to DXA, more accurate equations were established recently for middle-aged and older Japanese [113] and Caucasian [114] subjects.

Using DXA as a criterion measure, Leahy et al. developed prediction equations to compute %BF from SATs measured via B-mode US [52]. They built on previous experience with accurate measurements of SAT thickness [60]. In a sample of young adults, 52 women and 83 men, aged 18–29 y, they established the following equations [52]: for women, %BF = 17.95 + 0.28 × (abdominal SAT) + 0.54 × (medial calf SAT), whereas for men, %BF = 7.65 + 0.36 × (abdominal SAT) + 0.59 × (front thigh SAT). The agreement with the reference technique was very good, slightly better for men (SEE 1.9%, CE = −0.1%, LoA [−3.8%, 3.6%]) than for women (SEE = 3.0%, CE = 0.42%, LoA [−5.4%, 6.2%]) [52].

Gomez-Perez et al. derived a body fat prediction equation involving the mean of the abdominal SAT thicknesses determined on both sides of the umbilicus (at 5 cm from it, with a linear transducer placed horizontally) [115]. Their sample comprised 104 mostly obese and overweight adults (63F, 41M) aged 60.4 ± 6.25 y. For women, %BF = 19.16 + 0.74 × BMI + 0.50 × (abdominal SAT) resulted in SEE = 1.98%; for men, %BF = 6.19 + 0.59 × BMI + 3.26 × (abdominal SAT) ensured SEE = 2.01%. A single-site formula, based on the abdominal SAT at 1 cm above the umbilicus on the xiphoid–pubic line, was established also by Torgutalp et al. [116]. Their sample consisted of 45 adults of about 30 years of age, divided into a model development group (*n* = 31) and a validation group (*n* = 14). The formula established for women, FM = 17.221 + 0.276 × (abdominal SAT) × (height) had SEE = 3.6 kg, whereas for men, FM = 7.085 + 0.694 × (abdominal SAT) × (height) had SEE = 2.8 kg; here, height is expressed in m.

O’Neill et al. established formulas for body fat estimation in athletes via ultrasonography [63]. They used a GE Logiq E9 scanner (GE medical systems, Milwaukee, WI, USA) in musculoskeletal settings, with a GE L8-18i-SC probe working at 15MHz, to investigate a mixed gender group of 67 elite athletes aged 18–55 y. The search for potentially relevant anatomic locations started with seven ISAK sites, but multiple regression pointed out that only four of them had strong correlations with %BF measured via DXA, leading to the following formula: %BF = 1.846 + 0.467 × (sum of SATs at triceps, biceps, supraspinale, and front thigh). The SEE was 1.9%, the CE was negligible, and the LoA were [−3.7%, 3.7%] [63].

The athletic population was targeted also by Hyde et al. [117], who sought to establish a B-mode US protocol validated against the 3C model. Fifty-eight male collegiate football players were randomly divided into two groups of the same size: one for model development and another for cross-validation. They scanned the anatomical structures beneath the sites from the JP7 formula. Then, a linear regression equation was established between the sum of SAT thicknesses at all seven sites and %BF given by the 3C model: %BF = 6.194 + 0.096 × (sum of 7 SATs). The SEE of 2.97%, CE = 0.9%, and LoAs of about [−1.0%, 2.8%] demonstrated very good accuracy [117].

Smith-Ryan et al. turned to the original equations known from anthropometry that express body density in terms of SKFs [53]. They used the JP7 formula from anthropometry, established by Jackson and Pollock for men [69], and by Jackson, Pollock, and Ward for women [70], and replaced SKFs by SATs multiplied by two to express the thickness of a double layer of skin and subcutaneous fat.

Such an approach is clearly debatable because US measures the thickness of the uncompressed adipose tissue layer and, depending on the spatial resolution of the scan, it may also distinguish the thickness of the overlying skin, whereas an SKF measurement gives the thickness of a double layer of skin and fat squeezed by the jaws of the caliper. For reproducibility, the mechanical stress (force per unit area) exerted by the caliper is carefully calibrated (10 g/mm^2^). It is known that biological tissues are viscoelastic materials, and in anthropometry, the dynamics of SKF deformation have been thoroughly documented. For reliable results, it is recommended to deploy the caliper, wait for 2 s until the rapid elastic deformation is over, and take the SKF measurement within the next second [20]. Nevertheless, let us postpone this discussion to Section 5 and look at the results.

Just as in their previous work [75], Smith-Ryan et al. examined overweight and obese subjects (29 women and 22 men), aged 37.2 ±11.3 y [53]. Striving to improve the accuracy of US assessments of global adiposity, they used a high-resolution B-mode US system, NextGen Logiq-e (GE Healthcare, Waukesha, WI, USA) with a wide-band linear array probe (12L-RS) working at frequencies between 5 and 13 MHz. They adapted the JP7 formula to derive body density, and used the Siri equation to calculate %BF. In JP7, they replaced SKFs by twice the corresponding SATs measured without including the skin thickness. The criterion measure was the highly trusted 4C model developed by Wang et al. [17]; BV was determined by ADP, TBW was inferred from BIS, and bone mineral content was measured by DXA.

For the entire sample, the US assessment of %BF had a CE of 3.4%, SEE of 3.5%, and TE of 6.9%. For women, CE was 9.2% (a statistically significant overestimation, *p* < 0.001), SEE was 4.7%, and TE was 8.9%. For men, the results were encouraging: the CE was 0.7%, SEE was 2.4%, and TE was 3.7%, since they marginally satisfy the validity requirement of both the SEE and TE being at most 3.5% [118]. The reliability of the US-based estimation of %BF was deemed excellent. Based on two tests performed 24 to 48 h apart on each subject, the SEM was 0.94% for the entire sample, 0.99% for men, and 0.91% for women, which correspond to MDCs of 2.6%, 2,7%, and 2.5%, respectively. Also, the ICCs were 0.966 for all participants, 0.939 for men, and 0.994 for women. The authors concluded that their US-based approach ensured a fairly good body fat assessment for men, but not for women. Nevertheless, since the reliability of US tests was high for both women and men, it may accurately track changes in body composition [53].

The methodology proposed by Smith-Ryan et al. [53] was employed by Chandler et al., who explored the reliability of US-based body fat estimations when the image analysis was conducted in a semi-automatic way by means of MuscleSound^®^, an online software application that incorporates artificial intelligence (AI) algorithms [119]. Two technicians took triplicate readings on each subject at each of the seven sites involved in the JP7 equation. They uploaded all of their images into MuscleSound^®^ and verified that the fat–muscle interface was correctly identified by the AI software. All statistical measures, SEM < 0.54%, MDC < 1.49%, and ICC > 0.988 for %BF, indicated remarkable intratester reliability, for both testers, in both men and women. However, the intertester reliability was less impressive and much lower in men than in women (SEM = 1.56%, MDC = 4.32%, and ICC = 0.867 for %BF measures of men, and SEM = 0.64%, MDC = 1.77%, and ICC = 0.992 for women). The origin of this sex-dependence of the precision of SAT measurements by B-mode US remains to be clarified.

The accuracy of the B-mode US estimation of whole-body fat was further tested in athletes (ballet dancers, against ADP [47]) and in the general population (adult males, against the 3C model [50]). Chandler et al. evaluated the body composition of teenager ballet dancers (27F, 21M) via US and found very good agreement with ADP: a mean overestimation of %BF by 1%, SEE of 2.5%, and TE of 3.3%. The scrutiny of the data split by sex revealed that the accuracy of US was higher for males (CE = 0.1%, SEE = TE = 2.7%) than for females (CE = 1.6%, SEE = 2.1%, TE = 3.1%) [47].

Bradley et al. [120] looked at the reliability and validity of %BF assessment using JP7 and MuscleSound^®^ in a sample of 50 adults (25F, 25M) aged 18–39 y. They found high reliability (ICC = 0.997), but limited accuracy: compared to DXA, the CE of %BF estimates was −2.6% for the entire sample, −3.5% for men, and −1.6% for women.

In their study performed on resistance-trained men, Tinsley et al. [50] tested the accuracy of B-mode US combined with the JP7 formula applied under the assumption that SKF = 2 SAT. The US-derived FFM was fairly accurate, with CE values of 0.1 kg and 0.9 kg determined for the same sample at two different time points. Furthermore, the US-based estimate of body density was used to compute body volume (i.e., portable US served as a substitute for ADP) and BIA was used to evaluate TBW. The resulting field-based 3C model predicted the change in FFM with a mean difference of 0.2 kg and passed the equivalence test with the lab-based 3C model (the latter relied on ADP and BIS) [50].

Sullivan et al. [121] employed the 4C model criterion to investigate the accuracy of the B-mode US for body fat assessments by the JP7 equation from anthropometry assuming that SKF = 2 SAT. In their study group of mostly normal-weight adults, aged 23 ± 4.1 y (5F, 25M), US underestimated %BF: CE = −3.48%, SEE = 3.60%. Interestingly, the US assessment did not change much when the sum of SATs measured at the sites from JP7 [69,70] was replaced by seven times the average of SAT thicknesses measured at the standard IOC sites [41]: CE = −2.88%, SEE = 3.29% [121].

When US and DXA were compared regarding their ability to track the body composition during a 6-week intervention (supervised resistance training combined with a hypercaloric diet), their body fat estimates displayed higher correlations cross-sectionally than longitudinally [56]. These findings stress the importance of testing the capabilities of a technique to detect changes in body composition.

### 4.4. Ultrasound-Based Characterization of Subcutaneous Fat Patterning

Mechelli et al. tested the accuracy [122] and reliability [123] of SAT and muscle thickness measurements of the anterior thigh using a B-mode US. Their findings confirmed the validity of US versus MRI: for SAT thickness, CE = 0.55 mm and LoA = [−0.33, 1.43] mm, whereas for muscle thickness CE = −0.51 mm and LoA = [−2.85, 1.83] mm in a gender-balanced group of 20 middle-aged healthy subjects. However, the accuracy of US was unsatisfactory in perimuscular fascia thickness estimations: CE = 0.42 mm and LoA = [−0.97, 0.71] mm [122]. They also conducted a repeatability study on moderately active adults (12F, 12M). The intratester reliability, derived from two tests performed by the same observer, one week apart, was excellent for measurements of SAT (MDC = 1.3 mm, ICC = 0.99), very good for muscle (MDC = 3.6 mm, ICC = 0.96), and poor for fascia (MDC = 0.8 mm, ICC = −0.02). The intertester reliability was very good for SAT (MDC = 4.0 mm, ICC = 0.81) and muscle (MDC = 2.7 mm, ICC = 0.98) and fair for fascia (MDC = 0.4 mm, ICC = 0.70) [123].

Garcia-Herreros et al. [124] captured US images of the anterior thigh in 100 malnourished patients (40% M) by a portable B-mode US system with a linear probe, UProbe L6C Ultrasound Scanner (Guangzhou Sonostar Technologies, Guangzhou, China) working at 10 MHz. They compared the results of conventional image analysis and AI-based automatic image analysis using the PIIXMED^TM^ Ultrasound Imaging System (Dawako Medtech, Valencia, Spain). The two methods were in remarkable agreement: for SAT thickness CE = −0.04 mm, LoA = [−0.38, 0.30] mm, and ICC = 0.91, whereas for muscle thickness CE = 0.07 mm, LoA = [−0.11, 0.24] mm, and ICC = 0.96.

The accuracy of an A-mode US for SAT thickness mapping has been ascertained by direct measurements on cadavers [66] and by comparison with a B-mode US [67] (see also Section 4.1). In children, the precision of SAT thickness measurements performed with the BodyMetrix device differed between anatomic locations: the SEM was 0.6 mm for the triceps site, 1.0 mm for the subscapular site, and 1.2 mm for the supraspinale (suprailiac) site, whereas ICC was 0.91, 0.78, and 0.90, respectively [106]. In the elderly population, the reliability of SAT thickness measurements at the subscapular, abdomen, suprailiac, axilla, biceps, triceps, front thigh, and calf was excellent (ICC > 0.90), whereas for appendicular muscle thickness measurements it ranged from moderate to good (0.50 < ICC < 0.90) [125].

US stratigraphy of the subcutaneous tissues at the triceps, thigh, and abdomen was effective in tracking the efficacy of a nutritional and lifestyle intervention in adult men with overweight and obesity [126].

The IOC Medical Commission identified a need for precise and accurate portable instruments of body composition assessment in athletes [8], and US was among the most promising candidates [39,40,127]. The characterization of subcutaneous fat distribution by ultrasonography started from the standard ISAK sites because they were found relevant in SKF-based estimation of nutritional status [54]. Important milestones in this endeavor include (i) the refinement of the image acquisition procedure to mitigate measurement errors caused by fat compressibility and (ii) the development of a new software (US Tissue-FAT 3.3, Rotosport, Stattegg, Austria) conceived for an interactive, semi-automatic evaluation of the acquired US images [39,40]. Nevertheless, it turned out that the ISAK sites present several drawbacks for ultrasonography: (i) in US images of the ISAK trunk sites, it can be difficult to identify the SAT boundaries, (ii) just as in anthropometry, it takes months of technician training to ensure their accurate localization, and (iii) they are anchored to anatomical landmarks on the body surface, whereas US target sites could also be defined relative to features contained in the US image [40].

Therefore, the IOC Working Group on Body Composition, Health and Performance sought to identify more convenient sites according to the following requirements: (i) they need to sample the trunk, arms and legs; (ii) they should be located precisely by a novice technician after one hour of training; (iii) their positions should be described by distances expressed as fractions of the subject’s height; (iv) in their US image, anatomical structures should be easy to distinguish; and (v) the SAT thickness in their neighborhood should be roughly constant [41].

**Figure 5:**
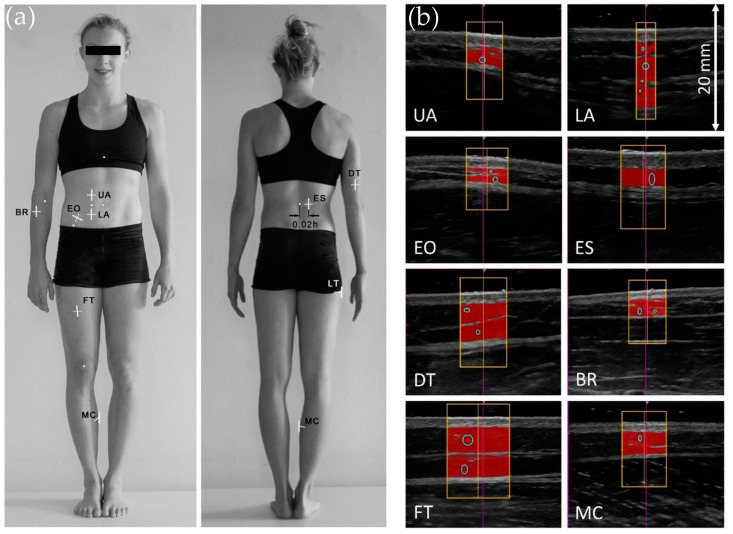
B-mode US assessment of subcutaneous fat patterning. (**a**) Sites proposed by IOC Working Group on Body Composition, Health and Performance [41]: upper abdomen (UA), lower abdomen (LA), external oblique (EO), erector spinae (ES), distal triceps (DT), brachioradialis (BR), front thigh (FT), medial calf (MC), and lateral thigh (LT). (**b**) Examples of US images processed by the US Tissue-FAT 3.3 software; in the region of interest centered on each site, SAT is highlighted in red. (This figure was adapted with permission from [128].)

Figure 5 represents sites that satisfy these criteria, as well as representative US images of the underlying anatomical structures [128]. Visual inspection of Figure 5a is insufficient for locating the measurement sites; the interested reader finds detailed instructions and a thorough analysis of representative US images in the work of Muller et al. [41]. Figure 5b illustrates the ability of the image analysis software to detect the borders of the SAT layer and fibrous structures embedded in the adipose tissue. The technician has the option to record the SAT thickness with or without the fibrous structures. These two options give similar results in the general populations, but in elite athletes at full training they lead to significantly different results [41].

The intertester reliability of SAT thickness measurements was much higher in the case of the IOC sites compared to the ISAK sites [40,55,129], an expected result, which confirms the importance of the criteria used for site selection. Nevertheless, the first methodological study, revealed that the EO site was difficult to mark precisely, especially in the case of subjects with a thick fat layer (to mark EO, one needs to palpate the subject to identify bony landmarks). Therefore, the working group proposed to replace EO with LT in the set of eight sites recommended for standardized SAT patterning using US [41,128,129].

The new standard served for testing athletes in practical settings [58,130,131], and it was adopted also outside the realm of sports medicine. It ensured highly reliable and accurate characterization of the subcutaneous adiposity of the general population, ranging from children [132,133], adolescents [134], and adults of diverse physiques [129]. Furthermore, standardized US provided valuable insights in the characterization of patients with anorexia nervosa [135,136,137]. Unlike conventional methods based on body proportions and body mass index, SAT patterning revealed that the sum of the eight SAT thicknesses (with fibrous structures included) was sufficiently high in half of the patients. Hence, they needed a different treatment protocol to avoid unnecessary fat gain while promoting muscle growth [136].

The IOC procedure worked also with the cost-effective, A-mode US (albeit with skin thickness included), to demonstrate that SAT thickness is not affected by changes in hydration status [59], a conclusion reinforced later by the B-mode US [138]. In contrast with DXA, neither resistance-exercise nor endurance-exercise was found to affect the sum of eight SAT thicknesses determined by standardized B-mode US [139].

US enables one to infer also the average subcutaneous fat thickness. Along with segmental body surface assessments, the mean SAT thickness was used to compute the SAT volume, in good agreement with MRI measurements, except for the thigh and upper arm [140]. SAT and muscle thickness assessments made on the anterior forearm enabled Abe et al. [141] to evaluate the subcutaneous fat mass of the forearm. Then, taking into account that adipose tissue comprises fat-free components in a proportion of 15% [142], they derived a prediction equation of DXA-derived appendicular fat-free adipose tissue. This is an important result because it enables an accurate estimation of the appendicular skeletal muscle mass, as a difference between the appendicular lean soft tissue mass and fat-free adipose tissue mass. Recent breakthroughs in this direction came from thorough body mapping and careful reliability tests [143,144,145]. Knowledge of the SAT mass enabled an indirect assessment of the visceral adipose tissue mass as the difference between total FM given by BIA and the SAT mass inferred from US-derived mapping of the SAT thickness. Moreover, prediction equations were derived for the total SAT mass based on merely three sites for women and four sites for men; these, combined with BIA, could be used to establish equations to estimate the mass of visceral adipose tissue in very good agreement with MRI [146].

Subcutaneous fat mapping by US is increasingly explored as a diagnostic tool. For example, Ata et al. [147] observed that vitamin D deficiency is associated with increased trochanteric SAT thickness, whereas the abdominal SAT was not significantly different between subjects with low versus normal levels of vitamin D. The authors suggest that vitamin D deficiency might be correlated with regional changes in the subcutaneous fat compartment. In another study, conducted on 158 pregnant women, Satish et al. [148] found that abdominal SAT was a predictor of perinatal adverse effects, including gestational hypertension and preterm delivery. The maternal SAT thickness was measured in their study at the cervix placenta view, taken along the linea alba, with the transducer in a mid-sagittal position above the symphysis pubis. Moreover, Budak et al. found that maternal abdominal SAT is a predictor of gestational diabetes mellitus [149]. In their study, the abdominal SAT thickness was measured at the intersection between the linea alba and the horizontal line that connects the uppermost points of the iliac crest. Anvery et al. found that US-derived measures of the subcutaneous fat thickness at several sites on the limbs and torso were correlated with the patients’ body image. The authors recommend high-resolution US to evaluate patients eligible for fat reduction procedures, as well as to track lipodystrophies caused by infections, autoimmune diseases, or medication [150]. Abe et al. investigated the relationship between US-derived SAT distribution and cardiovascular risk factors in postmenopausal women [151]. They measured the SAT thickness at six sites from the anterior and posterior aspects of the limbs and trunk [112] and used Bayesian linear regression to compute the ratio of high-density lipoprotein cholesterol to total cholesterol. The study of Abe et al. revealed that the abdominal SAT thickness, determined at 2–3 cm to the right of the umbilicus, was the most important predictor of dyslipidemia. Prasetyo et al. set out to derive a multivariable regression formula to predict the android/gynoid (A/G) ratio given by DXA from fat thicknesses determined by US [152]. Their attempt was successful only in women, and the resulting formula involved the UA and LA sites from Figure 5 along with the visceral fat thickness midway between the xiphoid and umbilicus. The A/G ratio, defined as the ratio of %BF measured in the abdominal (android) region and %BF measured in the gluteofemoral (gynoid) region, is a predictor of insulin resistance and atherosclerosis. Katz et al. used ultrasonography to evaluate the effectiveness of high-intensity focused electromagnetic fields for abdominal fat reduction [153]. Normal-weight obesity, a condition that entails increased risk of cardiovascular events, was investigated using an A-mode US in a sample of 184 women aged between 18 and 64 years. Cutoff values were established for the mean of SAT thicknesses measured at seven ISAK sites, which enabled an accurate classification of 85% of the NWO obesity cases [154]. Krauze et al. investigated the adipose tissue architecture of the thigh by ultrasonography and proposed to use it for sizing up the cardiovascular risk [155].

Efforts are being made to elaborate a standardized methodology for the US assessment of SAT and muscle mass in people with various conditions, including intensive care unit patients [156]. The clinical applications in patients with reduced mobility ask for US protocols that only involve anatomical locations on the frontal part of the body. Keeping this requirement in mind, Paris et al. developed, against DXA, a whole-body fat prediction equation that comprises sites on the anterior upper arm, abdomen, and front thigh [157]. Taken together, the works cited in the last two paragraphs of this section illustrate the increasing role of US in the evaluation of human body fatness in the clinics.

## 5. Discussion

Ultrasound provides multiple insights into body composition. The present review covered only a fraction of the field: measurements of the subcutaneous fat thickness and their uses in the evaluation of total body fat content in adults. The evaluation of pediatric body composition using US was charted by a recent scoping review [158]. Despite many works dedicated to these topics over the past decade, our study suggests that much remains to be explored.

The standardization of US measurements of SAT distribution has laid the foundation for accurate and reliable tracking of the amount of subcutaneous fat in healthy individuals and in patients with diverse conditions susceptible to impact the size and distribution of fat depots. The study of anorexia nervosa by Lackner et al. [136] is an interesting example and others are expected to follow.

The estimation of subcutaneous fat mass from a set of US-derived SAT thicknesses [143,146] may benefit from emergent techniques capable of three-dimensional (3D) characterization of body shape, such as 3D photonic scanning and smartphone applications [159].

Body fat percentage estimates derived from an A-mode US were valid in male athletes when the assessment was carried out using the BodyMetrix instrument and the proprietary 3-site Jackson and Pollock (JP3) equation from the BodyView software [51]. Therefore, this technique has been adopted in practice, e.g., for the evaluation of the body composition of professional football players [160]. Other sports might adopt this methodology, as well.

US-derived measures of whole-body fatness may become more accurate provided that population-specific prediction formulas will be developed in large and relatively homogeneous study groups meant to sample a certain population (e.g., athletic, overweight, obese). Cross-validation in an independent sample is an essential step of this process (see, e.g., [85,86]), which is often overlooked because of logistic constraints.

Anthropometry can provide hints for model development, but care should be taken with converting popular skinfold-based equations for ultrasonography. For example, many exciting advancements discussed in Section 4.3 are based on a questionable assumption, that a skinfold is about twice as thick as the corresponding uncompressed subcutaneous fat. It is no wonder that it gave rise to a debate [161,162]. Still, the question arises, why does such an approximation work? Does the elimination of the skin thickness compensate for the elastic deformation of the skinfold? Or is the formula inaccurate for the modern human phenotype and the approximation happens to correct it?

In an attempt to answer such questions, our team proposed to convert an anthropometric equation into a prediction formula for US-based estimation of whole-body fatness by substituting each SKF with the corresponding SAT multiplied by the experimentally determined value of the SKF/SAT ratio [49]. We converted 33 anthropometric equations and assessed their validity against ADP. Although none of them were accurate according to the criteria of Heyward and Wagner [118], some of them surpassed the accuracy of the built-in equations from BodyView in the case of obese and overweight subjects.

In the context of the JP7 formula, our approach resulted in a less valid estimation of %BF than by simply taking the skinfold thickness as twice the subcutaneous fat thickness. We used an A-mode US to measure the thickness of the fat layer beneath the skin; its resolution was insufficient to identify the skin–fat border, so the measured SAT also included the skin thickness. Nevertheless, despite completely ignoring the deformation of the skinfold, substituting twice SAT for SKF in the JP7 equation ensured reasonable accuracy. Further research will be needed to decipher this puzzle.

It seems reasonable to expect that a deeper understanding of skinfold viscoelasticity will clarify the technical aspects of converting well-established, population-specific anthropometric equations into US-based prediction formulas. It is essential to build large databases to characterize skinfold compressibility at diverse measurement sites and to quantify how much does it vary from one person to another. Recent advancements in US elastography could be useful in this respect [163]. Furthermore, comparative investigations of US-derived SAT thicknesses and SKF thickness measured using skinfold calipers [164,165,166,167,168,169,170,171] can clarify the relationship between ultrasonography and anthropometry. Such studies can be worthwhile since more than 100 anthropometric equations are known so far and they might serve as valuable resources for US-based estimation of whole-body fat content.

However, too much focus on the analogy with anthropometry might be delusive. Since skinfold compressibility depends on the measurement site, it can happen that sites that are most relevant for SKF-based estimation of whole-body fat are less relevant when US is used for the same purpose. Indeed, in Sloan’s pioneering study, multiple regression analysis indicated different pairs of sites for SKF-based and US-based equations for computing body density (thigh and subscapular for SKF; thigh and iliac crest for US) [111].

An interesting line of thought has been initiated by Bielemann et al. [87], who decided to include also muscle thickness values in their percent body fat prediction equation. On the other extreme, Takai et al. [172] relied completely on muscle thicknesses to derive regression equations for DXA-based FFM. The best one (SEE = 2 kg) expressed FFM in terms of limb muscle thicknesses multiplied by the corresponding limb lengths. Then, FM was obtained as the difference between BM and FFM. The present scoping review demonstrates that this area is underexplored; yet, although the relevance of the muscle compartment in body weight management is further supported by the observation of De Toni et al. [126], the variation in the triceps’ muscle thickness at 2 months was the most important predictor of the subsequent, long-term weight loss.

Another direction of future research is to derive whole-body fat prediction equations from scratch, focusing on anatomic locations that are convenient from the point of view of ultrasonography, as proposed by Müller et al. [41]. Such an approach would benefit from the superior reliability of SAT measurements at the eight standard IOC sites [129]. An important step in this direction has been performed by Sullivan et al. [121], and interesting developments are expected to come in the near future.

Finally, a promising area is the use of AI for body fat estimation using US. AI algorithms have revolutionized body composition analysis via computed tomography (CT). For example, the visceral fat area is commonly measured in an abdominal CT scan, in an axial section taken at the third lumbar vertebra. The manual segmentation of a single section takes about 15 min, whereas the same task is completed in about one second by a deep learning algorithm [173]. Important milestones and remaining challenges in the field of machine learning applied in CT-based body composition analysis are covered by a recent review [174]. In clinical US imaging, deep learning was applied, among others, for image segmentation and hardware optimization, e.g., to improve the image quality of handheld US scanners [175]. The fast pace of AI research will most likely lead to a wealth of fully automated segmentation software (some of them are already on the market and demonstrated high accuracy [119,124]) and improved whole-body fat prediction algorithms.

This study is not free from limitations. First, the search strategy comprised only five major databases. Moreover, for Google Scholar, we applied the search terms to document titles only because the full record search returned 17,500 hits—their screening was beyond our resources. Second, we only considered articles written in English. This inevitably caused some loss of breadth. For example, during the bibliography screening of a recent review paper [71], we identified an exciting study published in Portuguese [176]. The interested reader is referred to the narrative review written by Benatti de Oliveira et al. [71] for further details. Third, differences in study design, sample characteristics, and criterion methods may have contributed to the heterogeneity of the statistical measures of the accuracy of total body fat estimation using US. Fourth, differences in test protocols and statistical tools may have been responsible for part of the observed discrepancies between reliability studies.

In conclusion, the recent literature indicates that ultrasound is a non-invasive and relatively inexpensive technique with multiple applications in body composition research. This review described the uses of A-mode and B-mode ultrasound as tools for subcutaneous fat and whole-body fat assessment, a vast and dynamic research field, with exciting achievements, but also numerous challenges and underexplored areas.

The primary literature analyzed in this scoping review suggests that ultrasound is an accurate and reliable technique for measuring subcutaneous adipose tissue thickness. This is true for both A-mode and B-mode ultrasound, despite their different resolutions, of about 0.5 mm and 0.1mm, respectively. Therefore, ultrasound is recommended for subcutaneous fat measurements.

Assessments of total body fat content using ultrasound rely on prediction formulas optimized to ensure agreement with criterion measures. Their accuracy is less well established. The current literature suggests that prediction formulas are only valid for subjects whose phenotype was represented in the sample involved in model development. Thus, further research is warranted to derive accurate equations for diverse populations. There is a need for studies conducted on large samples, preferably with multicompartment models as criterion measures.

In contrast, the reliability of ultrasound-based estimation of the amount of whole-body fat was deemed very good, comparable to that of laboratory techniques. Therefore, ultrasound might be able to track changes in body fat content in longitudinal studies, either alone or in combination with other field-based methods. Future work will tell how well it can perform.

## Figures and Tables

**Table 1:** Original articles on the accuracy of %BF estimation by the A-mode US ^1^.

Reference	Population	Study Group	Equation ^2^	SEE (%)	TE (%)	CE [LoA] (%)	Criterion
Loenneke et al. [74]	athletic	gymnasts, 13F, age 20 ± 1 y	Bic1 JP3	3.6 3.9	6.7 4.9	3.4 5.7	DXA
Smith-Ryan et al. [75]	overweight and obese	20M, 27F age 37.6 ± 11.6 y	JP7	-	-	−4.7	3C
Muntean et al. [49]	general	107M, 94F age 31.6 ± 10.8 y	JP7	-	-	−4.8 [−14.2, 4.5] F −4.8 [−14.5, 5.0] M	ADP
Hendrickson et al. [76]	general	21M, 10F age 26.7 ± 3.9 y	JP3	-	-	−1.0 [−10.0, 8.0]	ADP
Totosy de Zepetnek et al. [77]	general	16M, 33F age 31.4 ± 10.7 y	JP7	-	-	−0.32 [−7.87, 7.22]	ADP
Wagner et al. [51]	athletes	22M, 23F age 20.1 ± 1.6 y	JP3	2.6	4.4	1.5 for M 4.7 for F	ADP
Kendall et al.	athletes FFM (kg)	23M age 24.6 ± 2.2 y		3.8 kg	7.2 kg	4 kg [−3.2, 11.3] kg	4C
Johnson et al. [78]	general	35M, 49F age 23 ± 4.7 y	JP7	0.7	-	−4.4 for M −3.7 for F	DXA
Johnson et al. [79]	general FFM (kg)	33M, 41F age 23.1 ± 4.9	JP7	-	-	0.37kg [−7.9, 8.7] kg	ADP
Baranauskas et al. [80]	general	33M, 43F age 22.08 ± 2.5 y	JP7 JP3 P3	- - -	- - -	−3.9 −3.6 −5.2	DXA
Olinto et al. [81]	general	23M age 30.1 ± 7.7 y	JP3	-	-	−9.6 [−17.1, −2.0]	DXA
Kang et al. [82]	general	105M age 20.01 ± 2.11 y	Bic1 S2 JP3 P3 NHCA4 FS4 DW4 JP7 P9	- - - - - - - - -	- - - - - - - - -	2.3 [−6.1, 10.7] −7.2 [−16.7, 2.3] −7.0 [−15.9, 2.0] −5.1 [−13.5, 3.2] −6.4 [−15.6, 2.8] −1.4 [−22.1, 19.3] −1.3 [−10.8, 8.2] −6.4 [−14.6, 1.8] −0.4 [−11.3, 10.4]	DXA
Lowry et al. [48]	elite, athletic and non-athletic	42M age 21.4 ± 2.9 y	Bic1 S2 JP3 P3 NHCA4 FS4 DW4 JP7 P9	3.67 4.8 4.03 3.25 3.9 4.86 3.82 3.55 4.55	7.66 4.72 4.08 3.93 4.16 11.34 7.11 3.69 6.5	6.54 −1.23 0.94 1.93 1.68 8.58 6.01 1.23 4.72	ADP
Ripka et al. [83]	adolescents	143M age 14.8 ± 1.5 y	JP7 new	- 1.45	- -	−9.37 0.45 [−4.25, 5.16]	DXA
Ripka et al. [84]	adolescents	71M, 34F age 14 ± 2 y M age 13 ± 2.3 y F	new	1.57	-	0.0 [−7.0, 7.0] F 0.2 [−5.4, 5.8] M	DXA
Pineau et al. [85]	athletes	100M; 62M cross-validation	new	1.6	-	−0.25 [−4.1, 3.6]	DXA
Pineau [86]	general	63M; 35M cross-validation	new	2.9	-	0.30 [−5.1, 5.7]	DXA
Bielemann et al. [87]	general	102M, 104F age 30 ± 8.1 y M age 31.9 ± 9.9 y F	new	-	-	0.5 [−6.8, 7.7] M 0.1 [−6.6, 6.7] F	ADP
Schoenfeld et al. [88]	general	20F age 22.4 ± 2.8 y	JP4	4.17	4.08	0.9 [−7.1, 8.9]	ADP
Bradley et al. [89]	general	29M age 18–25 y	JP3	-	-	0.0 [−5.3, 5.3]	ADP

^1^ Abbreviations: ADP—air displacement plethysmography; DXA—dual-energy X-ray absorptiometry; 3C—three-compartment model; FFM—fat-free mass; F—female; M—male; SEE—standard error of estimate; TE—total error; CE—constant error, the mean difference between the compared methods; LoA—limits of agreement, listed as [CE − 1.96 × SDD, CE + 1.96 × SDD], where SDD denotes the standard deviation of the differences between methods; CE is reported as the mean of the body fat estimate given by the evaluated technique minus that given by the reference method. ^2^ Acronyms of BodyView’s built-in equations tested for validity: Bic1—one-point biceps; DW4—4-site Durnin and Womersley; FS4—4-site Forsyth–Sinning; JP3, 4, 7—3, 4, 7-site Jackson and Pollock; NHCA4—4-site National Health Center of America; P3—3-site Pollock; P9—9-site Parrillo; S2—2-site Sloan.

**Table 2:** Original studies of the reliability of %BF assessment by the A-mode US ^1^.

Reference	Population	Study Group	Equation ^2^	SEM (%)	MDC (%)	ICC
Loenneke et al. [104]	general	students, 3F, 8M, age 22 ± 3 y	Bic1 JP3	- -	2.8 5.6	0.977 0.935
Smith-Ryan et al. [75]	general	overweight and obese, 27F, 20M, age 37.6 ± 11.6 y	JP7	2.2	6.1	0.980
Hendrickson et al. [76]	general	adults, 10F, 21M, age 26.7 ± 3.9 y	JP3 JP3 *	- -	- -	0.800 0.870
Chirita-Emandi et al. [105]	general	adults, 1F, age 31 y, 1M, age 24 y	JP3 JP3 *	0.78 0.45	- -	0.982 0.991
Chirita-Emandi et al. [106]	general	children, 20F, 20M, age 11.9 ± 3.7 y	JP3 *	0.94	-	0.954
Totosy de Zepetnek et al. [77]	general	16M, 33F age 31.4 ± 10.7 y	JP7	0.78	2.16	0.986
Miclos-Balica et al. [107]	general	adults, 63F, 81M, age 30.4 ± 10.1 y	JP7 JP7 * JP3 JP3 * P3 Bic1	1.06 1.24 1.52 1.76 1.57 2.54	2.95 3.43 4.21 4.87 4.34 7.05	0.979 0.972 0.954 0.938 0.955 0.964
Wagner et al. [51]	athletic	23F, age 19.6 ± 1.4 y 22M, age 20.6 ± 1.6 y	JP3 JP3 *	- -	1.80 -	0.993 0.987
Wagner and Teramoto [65]	general	32F, age 22.1 ± 1.1 y 48M, age 24.4 ± 1.6 y	JP3 * JP3 *	1.48 0.94	4.10 2.60	0.969 0.990

^1^ Abbreviations: F—female; M—male; SEM—standard error of measurement; MDC—minimal detectable change; ICC—intraclass correlation coefficient. ^2^ Acronyms of BodyView’s proprietary equations tested for reliability: Bic1—one-point biceps; JP3, 4, 7—3, 4, 7-site Jackson and Pollock; P3—3-site Pollock. * Asterisks denote intertester reliability, whereas the unmarked lines list the less favorable intratester reliability indicators.

## Data Availability

This article does not report original data.

## Supplementary Materials

The following are available online at https://www.mdpi.com/article/10.3390/life15020236/s1. Table S1: Search terms and the corresponding numbers of returned records.

## List of Abbreviations

2, 3, 4C	2, 3, 4-compartment model
%BF	percent body fat
ADP	air displacement plethysmography
AI	artificial intelligence
A-mode	amplitude-mode
B-mode	brightness-mode
BIA	bioelectrical impedance analysis
BMI	body mass index
Bic1	one-point biceps
BIS	bioelectrical impedance spectroscopy
BM	body mass
BR	brachioradialis
CE	constant error
CT	computed tomography
D	body density
DT	distal triceps
DW4	4-site Durnin and Womersley
DXA	dual-energy X-ray absorptiometry
EO	external oblique
ES	erector spinae
F	female
FFM	fat-free mass
FM	fat mass
FS4	4-site Forsyth–Sinning
FT	front thigh
GLP-1	glucagon-like peptide-1
HW	hydrostatic weighing
ICC	intraclass correlation coefficient
IOC	International Olympic Committee
ISAK	International Society for the Advancement of Kinanthropometry
JP3, 4, 7	3, 4, 7-site Jackson and Pollock
LA	lower abdomen
LoA	limits of agreement
LT	lateral thigh
M	male
MC	medial calf
MDC	minimal detectable change
MRI	magnetic resonance imaging
NHCA4	4-site National Health Center of America
P3	3-site Pollock
P9	9-site Parrillo
PRISMA	Preferred Reporting Items for Systematic Reviews and Meta-Analyses
S2	2-site Sloan
SAT	subcutaneous adipose tissue
SEE	standard error of estimate
SEM	standard error of measurement
SKF	skinfold
TE	total error
UA	upper abdomen
US	ultrasound
