# Supplementary material for: A Decade of Progress in Ultrasound Assessments of Subcutaneous and Total Body Fat: A Scoping Review
Source: Life (Basel). 2025 Feb 5;15(2):236. doi: 10.3390/life15020236 (PMC11856862; doi:10.3390/life15020236)
Supplement: Supplementary file 1 [file life-15-00236-s001.zip › life-3429472-supplementary.pdf]

life

MDPI

Supplementary Materials

for the Article

A Decade of Progress in Ultrasound Assessments of Subcutaneous and Total Body Fat: A Scoping Review

Monica Neagu and Adrian Neagu

This text provides further details on the systematic search conducted for the scoping study of ultrasound-based measurements of subcutaneous adipose tissue (SAT) thickness and the assessment of whole-body fat content in terms of SAT thicknesses determined at selected sites.

We searched for potentially relevant documents in PubMed, MEDLINE, Scopus, Web of Science, and Google Scholar. In the case of Google Scholar, the search was restricted to the document titles to keep the number of hits at a manageable level.

Although language restrictions were imposed only in the Scopus search, the search terms were elaborated in English, and therefore, the returned results were articles written in English with few exceptions, whereby the title and abstract were bilingual (English included), but the main text was written in a foreign language; those articles were not included in the review.

During the first four months of the study, each reviewer devised their search strategy individually, in an iterative manner. The search terms were compared and refined during periodic discussions of the obtained results. The final search terms were established in March 2024 and applied on a monthly basis until December 2024. They are listed in Table S1 for each database along with the corresponding number of hits as of 12/20/2024.

Table S1. Search terms and the corresponding numbers of returned records

Database (No. of records)	Search term
**PubMed** and **MEDLINE** (1002 results)	((((ultrasound[Title/Abstract]) OR (ultrasonography[Title/Abstract]) OR (sonography[Title/Abstract]) OR (echography[Title/Abstract])) AND (("body composition"[Title/Abstract]) OR ("subcutaneous fat"[Title/Abstract]) OR ("subcutaneous adipose tissue"[Title/Abstract]) OR ("body fat"[Title/Abstract]) OR ("fat-free mass"[Title/Abstract]) OR ("lean body mass"[Title/Abstract]))) NOT ((visceral[Title/Abstract]) OR (intramuscular[Title/Abstract]) OR (intracellular[Title/Abstract]) OR (liver[Title/Abstract]) OR (hepatic[Title/Abstract]) OR (pericardial[Title/Abstract]) OR (pelvic[Title/Abstract]) OR (renal[Title/Abstract]) OR (lung[Title/Abstract]) OR (chest[Title/Abstract]) OR (thyroid[Title/Abstract]) OR (tumor[Title/Abstract]) OR (carcinoma[Title/Abstract]) OR (fetal[Title/Abstract]) OR (preterm[Title/Abstract]) OR (premature[Title/Abstract]) OR (infant[Title/Abstract]) OR (livestock[Title/Abstract]) OR (animal[Title/Abstract]) OR (carcass[Title/Abstract]) OR (goat[Title/Abstract]) OR (sheep[Title/Abstract]) OR (ewe[Title/Abstract]) OR (horse[Title/Abstract]) OR (calves[Title/Abstract]) OR (cow[Title/Abstract]) OR (pig[Title/Abstract]) OR (rat[Title/Abstract]) OR (mouse[Title/Abstract]) OR (mice[Title/Abstract]))) AND (("2014/01/01"[Date - Publication] : "2024/12/30"[Date - Publication]))
**Scopus** (1477 results)	TITLE-ABS-KEY ((ultrasound OR ultrasonography OR sonography OR echography) AND ("body composition" OR "subcutaneous fat" OR "subcutaneous adipose tissue" OR "body fat" OR "fat-free mass" OR "lean body mass") AND NOT (visceral OR intramuscular OR intracellular OR liver OR hepatic OR pericardial OR pelvic OR renal OR lung OR chest OR thyroid OR tumor OR *carcin* OR cancer OR fetal OR preterm OR premature OR infant OR livestock OR animal OR carcass OR goat OR sheep OR ewe OR horse OR calves OR cow OR pig OR rat OR mouse)) AND PUBYEAR > 2013 AND PUBYEAR < 2025 AND (LIMIT-TO (DOCTYPE , "ar") OR LIMIT-TO (DOCTYPE , "re") OR LIMIT-TO (DOCTYPE , "cp")) AND (LIMIT-TO (LANGUAGE , "English"))
**Web of Science** (1052 results)	(TI=((ultrasound OR ultrasonography OR sonography OR echography) AND ("body composition" OR "subcutaneous fat" OR "subcutaneous adipose tissue" OR "body fat" OR "fat-free mass" OR "lean body mass") NOT (visceral OR intramuscular OR intracellular OR liver OR hepatic OR pericardial OR pelvic OR renal OR lung OR chest OR thyroid OR tumor OR fetal OR preterm OR premature OR infant OR livestock OR animal OR carcass OR goat OR sheep OR ewe OR horse OR calves OR cow OR pig OR rat OR mouse)) AND PY=(2014-2024))<br>OR<br>(AB=((ultrasound OR ultrasonography OR sonography OR echography) AND ("body composition" OR "subcutaneous fat" OR "subcutaneous adipose tissue" OR "body fat" OR "fat-free mass" OR "lean body mass") NOT (visceral OR intramuscular OR intracellular OR liver OR hepatic OR pericardial OR pelvic OR renal OR lung OR chest OR thyroid OR tumor OR fetal OR preterm OR premature OR infant OR livestock OR animal OR carcass OR goat OR sheep OR ewe OR horse OR calves OR cow OR pig OR rat OR mouse)) AND PY=(2014-2024))<br>OR<br>(AK=((ultrasound OR ultrasonography OR sonography OR echography) AND ("body composition" OR "subcutaneous fat" OR "subcutaneous adipose tissue" OR "body fat" OR "fat-free mass" OR "lean body mass") NOT (visceral OR intramuscular OR intracellular OR liver OR hepatic OR pericardial OR pelvic OR renal OR lung OR chest OR thyroid OR tumor OR fetal OR preterm OR premature OR infant OR livestock OR animal OR carcass OR goat OR sheep OR ewe OR horse OR calves OR cow OR pig OR rat OR mouse)) AND PY=(2014-2024))
**Google Scholar** (176 results)	allintitle: (ultrasound OR ultrasonography OR sonography OR echography) AND ("body composition" OR "subcutaneous fat" OR "subcutaneous adipose tissue" OR "body fat" OR "fat-free mass" OR "lean body mass") -visceral -intramuscular -intracellular -liver -hepatic -pericardial -pelvic -renal -lung -chest -thyroid -tumor -fetal -preterm -premature -infant -livestock -animal -carcass -goat -sheep -ewe -horse -calves -cow -pig -rat -mouse -mice
